# Tibetan-PASEM: Phonology-Aware Speech Evidence Matching for Low-Resource Tibetan Written-Query Keyword Spotting

**DOI:** 10.3390/s26165107

**Published:** 2026-08-12

**Authors:** Yanze Guo, Xingmeng Guo, Zengguang Li, Jiaxin Song, Yuyang Gong, Guanyu Li

**Affiliations:** 1 China Institute of National Information Technology, Northwest Minzu University, Lanzhou 730030, China; y252430973@stu.xbmu.edu.cn (Y.G.);; 2 Key Laboratory of China’s Ethnic Languages and Culture Computing, Ministry of Education, Lanzhou 730030, China; 3 Data Intelligence Laboratory for Tibetan Plateau Human-Environment Systems, Lanzhou 730000, China

**Keywords:** user-defined keyword spotting, written-query keyword spotting, Tibetan speech processing, low-resource speech, phonological query representation, acoustic evidence matching, false-alarm control

## Abstract

**Highlights:**

**What are the main findings?**

- Tibetan-PASEM matches structured Tibetan written-query evidence with local acoustic-window evidence through latent-window aggregation and top-ranked local verification.
- A recording-content audit yields a corrected 213,128-pair Querybank198 evaluation with no identified development-to-held-out overlap in acoustic identity, source-qualified speaker, or session proxy.

**What are the implications of the main findings?**

- The method provides strong discrimination for training-exposed queries and a diagnostic view of query-specific false triggers.
- Transfer to held-out written queries remains partial and seed-sensitive, defining the primary target for further model and protocol development.

**Abstract:**

Low-resource written-query keyword spotting detects a text-specified target in speech without spoken enrollment or full automatic speech recognition. We present Tibetan Phonology-Aware Speech Evidence Matching (Tibetan-PASEM), a method that encodes graphemic, approximate phonological, and dialect-related query information, matches it with local acoustic windows, aggregates window-level evidence, and applies a validation-selected operating point. Querybank198 contains 198 Tibetan written queries derived from four public speech resources. A complete-file and decoded pulse-code modulation (PCM) audit produced a corrected 213,128-pair evaluation with no identified recording-content, source-qualified speaker, or session-proxy overlap across the development-to-held-out boundary. In a three-seed corrected-index re-evaluation, the acoustically strengthened PASEM (PASEM-AS) achieved F1 scores of 66.16 ± 2.14% on seen_test, 26.51 ± 3.42% on unseen_test, and 72.10 ± 1.39% on the confusable-negative diagnostic condition, which comprises 20 training-exposed query forms and is used for negative-set analysis rather than difficulty ranking. Under the original pre-correction pair index, PASEM with transfer regularization (PASEM-TR) increased the unseen_test F1 from 25.80 ± 2.71% to 35.25 ± 4.17% and the Recall from 18.48 ± 2.98% to 33.85 ± 8.22%; the corresponding seed-level 95% confidence intervals were 24.89–45.61% and 13.43–54.27%. These results establish strong familiar-query discrimination, an audited fixed-threshold evaluation protocol, and partial, seed-sensitive transfer to held-out written queries.

## 1. Introduction

Keyword spotting (KWS) determines whether a target expression occurs in speech without producing a complete transcript [1,2,3,4,5]. User-defined and open-vocabulary KWS extend this setting by allowing the target expression to be specified after model training [6,7,8,9,10,11,12,13]. Within Querybank198, this post-training query scenario is operationalized by unseen_test, which contains written query forms excluded from model training and is treated as a held-out transfer objective rather than an assumed deployment capability. This study considers a written-query setting in which a user supplies a Tibetan written form rather than a spoken enrollment example.

Low-resource written-query KWS is challenging because query-side information is textual, whereas the matching evidence is acoustic. Existing user-defined KWS methods differ in whether they use fixed keyword inventories, spoken enrollment, written queries, audio queries, phoneme-level supervision, or audio–text agreement objectives [6,7,8,9,10,11,12,13,14,15,16,17]. These assumptions are not interchangeable in Tibetan settings, where large transcribed speech corpora, complete pronunciation resources, and user-recorded enrollment examples remain limited. More broadly, connectionist temporal classification (CTC) [18], Conformer and attention-based sequence modeling [19,20], self-supervised speech representation models such as wav2vec 2.0 and Hidden-Unit BERT (HuBERT) [21,22], and model-compression methods [23] provide established components for low-resource acoustic modeling and transfer.

Three factors are particularly relevant to Tibetan written-query KWS. First, the available Tibetan speech resources are comparatively limited and heterogeneous, making protocol definition and reproducibility important [24,25,26,27]. Second, dialect-related surface realization, speech rate, and local context can alter the acoustic evidence associated with a written query [28,29]. Corpus-specific descriptions and broader under-resourced speech context further motivate explicit protocol reporting [30,31,32]. Third, short Tibetan expressions may share written components, syllables, codas, approximate phonological cues, or local acoustic patterns with non-target speech, increasing false-trigger risk [9,12,15]. Evaluation should therefore report fixed-threshold false-alarm and missed-detection behavior in addition to pooled detection accuracy.

This paper presents Tibetan Phonology-Aware Speech Evidence Matching (Tibetan-PASEM), a method for segment-level Tibetan written-query KWS. The method constructs structured query evidence from normalized graphemic forms, approximate phonological cues, and dialect-related syllable features; matches this evidence with local acoustic windows; aggregates window-level matching evidence; and produces a pair-level posterior through top-ranked local verification. Querybank198 separates familiar-query detection, held-out written-query transfer, and query-specific false-trigger diagnosis under a common validation-selected operating-point policy. The experiments evaluate pre-constructed query–speech pairs using approximately 2.0 s acoustic windows [10,12,15].

This study makes four contributions. First, Tibetan-PASEM formulates Tibetan written-query KWS as phonology-aware evidence matching between structured query representations and local acoustic windows. Second, it introduces latent-window aggregation with top-ranked local verification, enabling pair-level training without manually annotated keyword boundaries. Third, it evaluates graphemic, approximate phonological, and dialect-related query evidence under a common acoustic encoder and operating-point policy. Fourth, it provides an audited Querybank198 protocol-and-evaluation package, including original and corrected pair indexes, recording-content and split-overlap audits, same-query controls, threshold-selection scripts, archived Tibetan-PASEM pair-level scores, and score-alignment records [24,25,26,27].

The evaluation includes the Stage 1–4 ablation chain, query-view sensitivity analysis, a same-query ordinary-negative control, query-level error visualization, fixed-budget reference implementations under declared input assumptions, and a corrected development-to-held-out audio-disjoint re-evaluation. The grapheme-only configuration serves as the direct written-text acoustic matching (Direct-ST) internal control rather than as a separate method.

The remainder of this paper is organized as follows. Section 2 describes the task definition, Querybank198 protocol, query evidence representation, acoustic-window encoder, latent-window evidence matching, controlled instantiations, teacher-guided regularization, implementation details, and threshold-selection policy. Section 3 reports the stage-wise results, fixed-threshold operating-point behavior, same-query and confusable-negative analyses, query-view sensitivity analysis, and external-reference comparisons. Section 4 discusses the formal evidence-matching contribution, approximate phonological evidence, split-specific behavior, false-alarm control, evaluation limitations, and implications for low-resource written-query KWS. Section 5 concludes the paper.

## 2. Materials and Methods

### 2.1. Task Definition

The task is defined as segment-level Tibetan written-query KWS. Given a written Tibetan query q and a recorded speech segment x, the system estimates whether the expression specified by q occurs in x. The pair-level label is y∈0,1, where y=1 indicates that the query is present and y=0 indicates non-target speech. The system does not require a spoken enrollment example or a full automatic speech recognition (ASR) transcript. Instead, it produces a query-conditioned posterior and a thresholded decision:

$$\hat{y}\left(q,x\right)=I\left[P_{\theta }\left(Y=1|q,x\right)\geq \tau \right],$$

where Pθ(Y=1|q,x) is the query-conditioned acoustic matching posterior and τ is selected on the validation split before held-out testing. This task definition follows written-query and user-defined KWS settings in which the target is specified independently of the evaluation speech [6,7,8,9,10,11,12,13,14]. The present protocol evaluates pre-constructed query–speech pairs and segment-level decisions. Table 1 summarizes the notation and main components used throughout the Tibetan-PASEM formulation.

### 2.2. Querybank198 Pair Construction and Development-to-Held-Out Audio-Disjoint Evaluation

Querybank198 is a derived evaluation protocol constructed from publicly obtainable Tibetan speech resources, including TIBMD@MUC, XBMU-AMDO31, Tibetan Greetings, and NICT-Tib1 [24,25,26,27]. These resources cover Amdo, Kham, and U-Tsang/Lhasa-related speech recorded under different corpus, speaker, and acoustic conditions. Querybank198 releases protocol metadata and indexed pair files rather than redistributing the source audio. The protocol materials can be mapped to locally obtained copies of the four source resources through the provided path-mapping configuration.

The protocol distinguishes the unit used for keyword-spotting evaluation from the unit used for split-independence auditing. The evaluation unit is an indexed query–speech pair. A single speech recording can be evaluated against multiple written queries and can therefore generate multiple positive or negative pairs. Consequently, the number of query–speech pairs is not equivalent to the number of independent speech recordings. The recording unit used for the independence audit is the complete referenced waveform-audio (WAV) file. The approximately 2.0 s acoustic windows used by Tibetan-PASEM are generated from these referenced recordings during feature construction and are not treated as independent stored recordings or split-assignment units.

The original Querybank198 index contained 198 written Tibetan query entries and 214,644 indexed query–speech pairs: 127,536 pairs in seen_train, 28,848 in seen_cv, 43,026 in seen_test, 2706 in unseen_test, and 12,528 in the legacy hardest_test file. The seen_train partition provides model-training pairs for 99 training-exposed query forms, while seen_cv uses the same query role for validation and operating-point selection. The seen_test condition evaluates training-exposed query forms on held-out speech, and unseen_test evaluates 79 written query forms excluded from model training after coverage filtering. After development-to-held-out audio correction, unseen_test contains 2697 indexed pairs, including 451 positive and 2246 negative pairs. This corresponds to an average of 5.71 positive pairs per held-out query form, although the pair distribution is not assumed to be uniform across queries.

The legacy hardest_test file comprises 20 of the 99 training-exposed query forms and evaluates them against a targeted confusable-negative construction. In seen_train, these 20 query forms account for 38,240 query–speech pairs, including 9560 positive pairs, representing 30.0% of all seen_train pairs. This corresponds to an average of 478 positive pairs per query, compared with 322.06 positive pairs per query across all 99 seen queries. The revised protocol therefore terms this evaluation the confusable-negative condition and interprets it as a targeted diagnostic of negative-set composition rather than an ordered difficulty tier. These evaluation roles follow user-defined and open-vocabulary KWS settings in which familiar-query detection, held-out written-query transfer, and query-specific false-trigger behavior may differ [6,7,8,9,10,11,12,13,15].

The protocol is not source-corpus-disjoint. Recordings from the same four public resources may contribute to different query roles so that query-role comparisons are not confounded with a complete change of corpus. Split independence is instead enforced across the development-to-held-out boundary at the recording-content, source-qualified speaker, and derived session-proxy levels. The development partitions comprise seen_train and cleaned seen_cv. The held-out evaluation conditions comprise seen_test, unseen_test, and the confusable-negative condition.

Recording-content independence was audited using canonical file paths and content hashes. Complete-file Secure Hash Algorithm 256 (SHA-256) values were computed for all 91,988 referenced WAV paths. Decoded pulse-code modulation (PCM) SHA-256 values were additionally computed where PCM decoding was supported, allowing files with different names, paths, or container headers but identical decoded acoustic samples to be identified. Files for which decoded-PCM hashing was unavailable remained covered by complete-file hashing and were recorded separately in the audit manifest. Two referenced recordings were assigned to the same acoustic-identity group when either their complete-file hashes or decoded-PCM hashes were identical.

The corrected protocol retains seen_train unchanged. From seen_cv, all query–speech pairs associated with an acoustic-identity group present in seen_train were removed. From seen_test, unseen_test, and the confusable-negative condition, all pairs associated with an acoustic identity present in seen_train or cleaned seen_cv were removed. Every query–speech pair associated with an excluded recording was removed jointly, irrespective of its query identifier or pair label. This procedure removed 1516 pairs and produced a 213,128-pair evaluation: 127,536 pairs in seen_train, 27,577 in seen_cv, 42,843 in seen_test, 2697 in unseen_test, and 12,475 in the confusable-negative condition.

Speaker independence was audited using source-qualified speaker identifiers formed by combining the source-resource identifier with the corpus-specific speaker identifier. No source-qualified speaker is shared between seen_train or cleaned seen_cv and any held-out evaluation condition. The four source resources do not provide a consistent common recording-session identifier. Session behavior was therefore audited using conservative speaker-scoped session proxies derived from explicit corpus path structure, recording-condition folders, dated recording groups, and corpus subfolders. These proxies also show zero overlap across the development-to-held-out boundary. The corrected protocol is recording-content-disjoint under the complete-file and decoded-PCM identity criteria, speaker-disjoint, and proxy-session-disjoint across the development-to-held-out boundary. Official recording-session independence is not inferred beyond the metadata supplied by the original resources.

**Figure 1:**
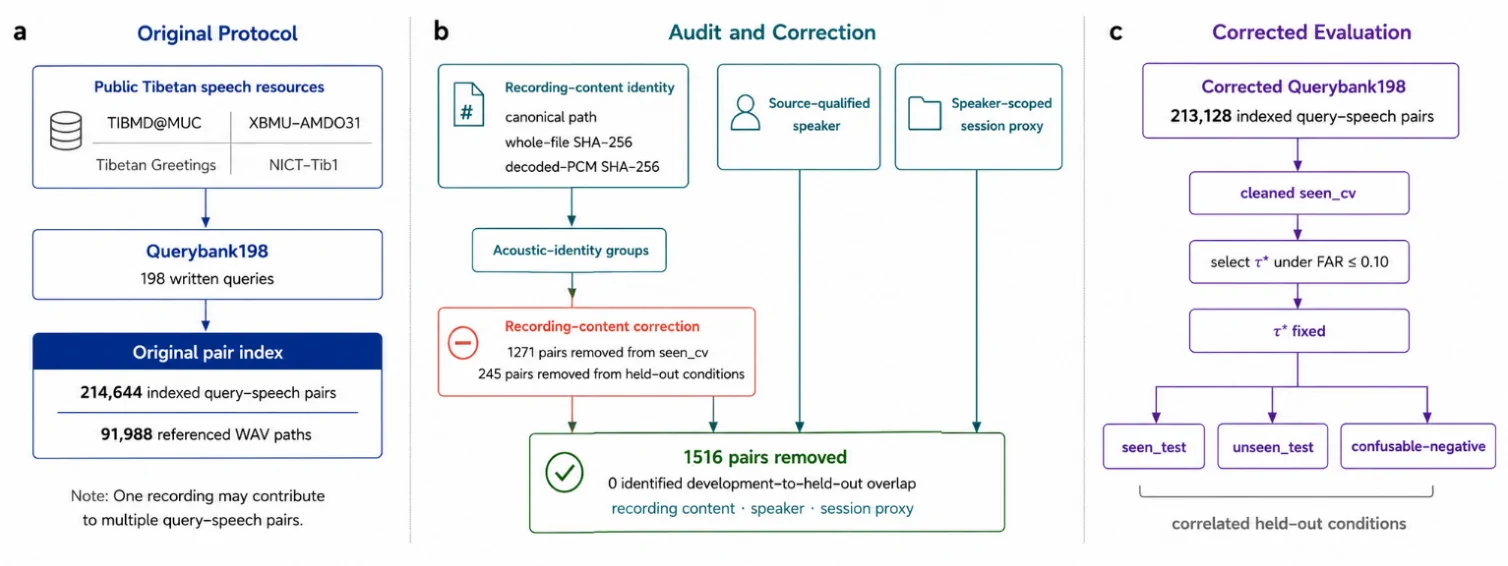
Querybank198 audit and correction. (**a**) Original query–speech pair index derived from four public Tibetan speech resources. (**b**) Recording-content correction based on canonical paths and whole-file/decoded-PCM hashes, together with parallel source-qualified speaker and session-proxy audits. (**c**) Corrected 213,128-pair protocol using an operating threshold selected on cleaned seen_cv and fixed for the three correlated held-out conditions. Abbreviations: WAV, waveform audio; PCM, pulse-code modulation; SHA-256, Secure Hash Algorithm 256; FAR, false alarm rate.

The three held-out conditions partially reuse held-out recordings and speakers. They are therefore interpreted as correlated condition-specific evaluations of seen-query detection, unseen written-query transfer, and confusable-negative behavior rather than as three statistically independent audio test sets. This held-out-to-held-out reuse does not cross the development-to-held-out boundary. Threshold selection and fixed held-out application follow the policy defined in Section 2.8 [10,12,15]. Figure 1 summarizes the original pair index, the recording-content correction and parallel speaker/session-proxy audits, and the corrected fixed-threshold evaluation.

The complete removal manifest, whole-file and decoded-PCM hash records, original and cleaned pair indexes, speaker and session-proxy overlap matrices, threshold-selection records, and score-to-pair alignment audits are provided in the Supplementary Materials. These materials support reconstruction of the Querybank198 protocol and the corrected development-to-held-out evaluation when the four source speech resources are obtained locally [24,25,26,27,33]. The released components and their supported reproduction roles are summarized in Table 2.

Protocol reconstruction consists of local path configuration, pair-index alignment, acoustic-identity correction, and score-level fixed-threshold evaluation. The release supports protocol construction, overlap auditing, threshold selection, and metric recalculation. Complete end-to-end model-training code is outside the current release scope.

### 2.3. Query Evidence Representation

Each Tibetan written query is represented by three complementary evidence views. The graphemic view g(q) preserves the normalized written Tibetan form. The approximate phonological view p(q) is obtained from inherited lexical or syllable-level entries when available and from a rule-based Wylie-style fallback when no inherited entry is available. The dialect-related feature vector d(q) summarizes conservative syllable- and pronunciation-related cues, including syllable count, onset/rime/coda-like patterns, syllable-structure indicators, and available Amdo, U-Tsang/Lhasa-related, or Kham source indicators. This design follows phoneme- and phonology-aware KWS, where pronunciation-related evidence can help distinguish short and acoustically confusable keywords [9,12,15] while reflecting the dialectal diversity of Tibetic speech varieties [28,29].

The query encoder contains a character-token mean encoder, a phonological-token mean encoder, and a linear projection for handcrafted dialect-related features. The three views are concatenated and projected into a shared query evidence embedding:

(1) $$\;e_{q}=W_{q}\left[E_{g}\left(g\left(q\right)\right);E_{p}\left(p\left(q\right)\right);W_{d}d\left(q\right)\right],\;\;\;\;\;\;$$

where $E_{g}\left(\cdot \right)$ and $E_{p}\left(\cdot \right)$ encode graphemic and approximate phonological evidence, $W_{d}d\left(q\right)$ projects dialect-related features, and $W_{q}$ maps the concatenated evidence into the shared matching space.

The three query views are used as structured query-side matching evidence. The graphemic view preserves the normalized written form, while approximate phonological cues add pronunciation-related information from inherited lexical entries, syllable-level entries, and rule-based Wylie-style fallback mappings. The dialect-related feature vector summarizes conservative syllable and source indicators. Table 3 specifies the source category used for each approximate phonological representation. This design fits the low-resource setting, where complete dialect-specific pronunciation dictionaries are unavailable, but lightweight pronunciation metadata can improve discrimination among short and confusable written queries [9,12,15,24,25,26,27,28,29].

### 2.4. Acoustic-Window Encoder

The recorded speech input is processed as utterance-level and candidate-window acoustic evidence. Audio is converted to 16 kHz mono speech, and 80-dimensional log-Mel filterbank features are extracted using a 25 ms frame length and a 10 ms frame shift. Features are normalized by per-dimension mean and standard deviation. Each speech recording is divided into approximately 2.0 s acoustic windows with a 0.8 s shift. These windows provide the local acoustic evidence units used for query-conditioned segment-level matching.

For the formal Querybank198 evaluation, each acoustic window is encoded by a convolutional neural network (CNN)-Conformer encoder. Conformer-style architectures combine self-attention with convolutional modeling and are suitable for capturing both global and local dependencies in speech sequences [19]. In this work, the CNN-Conformer encoder contains convolutional subsampling, sinusoidal positional encoding, four Conformer blocks, four attention heads, a feed-forward dimension of 768, and a depthwise convolution kernel size of 15. The output sequence is mean-pooled into an acoustic-window evidence embedding $h_{i}$:

$$h_{i}=Pool\left(CNN-Conformer\left(F_{i}\right)\right),$$

where $F_{i}$ denotes the log-Mel feature sequence of the i−th acoustic window. The CNN-Conformer encoder supplies the local acoustic-window evidence used by the posterior matching function. Its convolutional and self-attention components provide local and longer-range speech representations for query-window matching [19,20].

### 2.5. Latent-Window Probabilistic Evidence Matching

Tibetan-PASEM uses a latent-window evidence-matching formulation. The latent variable Z denotes the candidate acoustic window that contains the written query. The model estimates local query–window evidence, summarizes the window-level matching logits, and produces the final pair-level posterior through top-ranked local verification. This design matches the Querybank198 supervision format, where pair-level labels are available without manually annotated keyword boundaries.

Using the acoustic-window embedding hi defined in Section 2.4 and the query embedding eq defined in Section 2.3, the model estimates a local query–window match probability:

πi(q,x)=σ(fm([hi;eq;hi⊙eq;|hi−eq|])),

where ⊙ denotes elementwise multiplication, [;] denotes concatenation, and πi(q,x) measures the local compatibility between the written query and the i−th acoustic window. The keyword location remains latent because Querybank198 provides pair-level labels without manually annotated keyword boundaries.

The matcher aggregates the window-level logits through normalized log-sum-exp:

s_ret(q,x) = log(Σ_{i∈W(x)} exp(l_i)) − log|W(x)|,

where W(x) is the candidate-window set and $l_{i}$ is the pre-sigmoid matching logit. This corresponds to γ = 1.0 in the generalized temperature-controlled formulation and provides pair-level matcher supervision without keyword-boundary annotations.

For local verification, the candidate windows are ranked by their matching logits, and the five highest-scoring windows are retained. Each selected window is represented by its acoustic embedding and corresponding matching logit. These top-five representations are concatenated with the query-side feature vector d(q):

$$u\left(q,x\right)=\left[h_{i_{1}},l_{i_{1}};\ldots ;h_{i_{5}},l_{i_{5}};d\left(q\right)\right].$$

The local verifier is a multilayer perceptron with two hidden layers of 256 and 128 units. The first hidden layer is followed by sigmoid linear unit (SiLU) activation, dropout with a rate of 0.1, and layer normalization; the second hidden layer uses SiLU activation and dropout before the scalar output:

$$s_{\theta }\left(q,x\right)=f_{\phi }\;\left(u\left(q,x\right)\right),$$

$$P_{\theta }\left(Y=1|q,x\right)=\sigma \;\left(s_{\theta }\left(q,x\right)\right).$$

**Figure 2:**
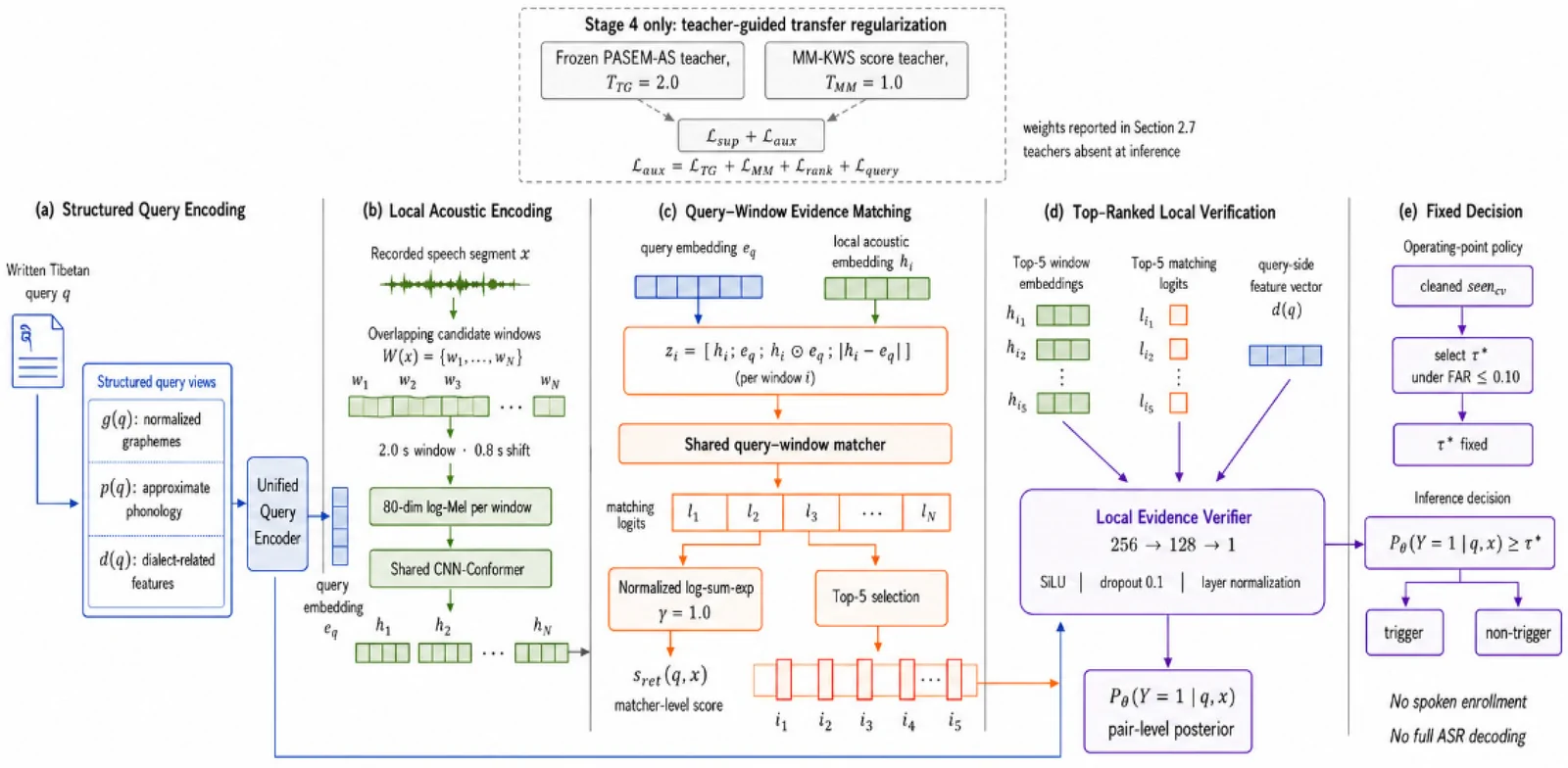
Tibetan-PASEM architecture. (**a**) Three structured query views are encoded into a shared written-query representation. (**b**) Overlapping speech windows are represented by 80-dimensional log-Mel features and a shared CNN-Conformer. (**c**) Query–window interactions produce matching logits used for matcher-level log-sum-exp scoring and top-five selection. (**d**) The selected window embeddings and logits, together with d(q), are processed by the local verifier to produce the pair-level posterior. (**e**) A validation-selected threshold produces the trigger decision. Dashed arrows denote Stage 4-only training signals. CNN, convolutional neural network.

The reported implementation therefore incorporates matcher evidence through the selected acoustic-window embeddings and their matching logits. It does not use manually specified retrieval–verification posterior coefficients. The same top-five selection rule, verifier architecture, and aggregation setting are used across the reported random seeds and evaluation conditions. Figure 2 summarizes the shared inference pathway and the Stage 4-only training signals. The inference model uses the structured query encoder, acoustic-window encoder, query–window matcher, top-five local verifier, and validation-selected operating threshold; the two teacher signals are absent at inference.

### 2.6. Controlled Ablation Instantiations of Tibetan-PASEM

The four stages are controlled instantiations of the same Tibetan-PASEM evidence-matching framework. Each instantiation enables one evidence source or training constraint while preserving the same Querybank198 data and evaluation protocol.

Stage 1, denoted PASEM-R, uses query-conditioned candidate retrieval only. It tests whether written-query evidence can retrieve plausible acoustic candidates.

Stage 2, denoted PASEM-RV, adds phonology-aware local verification. It tests whether top-ranked local acoustic evidence and query-side phonological features improve decision reliability under false-alarm pressure.

Stage 3, denoted PASEM-AS, introduces acoustically strengthened joint matching. It tests whether stronger acoustic-window evidence improves seen-query and confusable-negative discrimination.

Stage 4, denoted PASEM-TR, introduces teacher-guided transfer regularization during training. It is included to evaluate how auxiliary teacher information affects held-out written-query transfer under the same Querybank198 protocol.

The controlled instantiations measure how each evidence source changes performance on seen-query detection, unseen written-query transfer, and confusable-negative evaluation.

### 2.7. Teacher-Guided Transfer Regularization

Stage 4/PASEM-TR initializes the student from the selected Stage 3/PASEM-AS checkpoint and applies two fixed training-time signals. The first is the frozen PASEM-AS model defined in Section 2.3, Section 2.4 and Section 2.5, including the structured written-query encoder, CNN-Conformer acoustic-window encoder, query–window matcher, and local verifier. It is fitted on seen_train, selected on seen_cv, contains approximately 4.68 million parameters, and is used with TTG = 2.0.

The second signal consists of archived pair-level probabilities generated by the MM-KWS-style reference implementation described in Section 3.7 [12]. The reference model is fitted on seen_train and selected on seen_cv. Its archived probabilities are used as fixed score-level targets with TMM = 1.0; its architecture, internal embeddings, and parameters are not incorporated into the Tibetan-PASEM student. The effective reference configuration, fitting schedule, checkpoint-selection rule, archive-status disclosure, and score-generation audit summary are reported in Table S2 and File S1.

The Stage 4 objective is

$$L_{Stage4}=1.00L_{sup}+0.35L_{TG}+0.25L_{MM}+0.10L_{rank}+0.05L_{query},$$

where $L_{sup}$ is the supervised binary KWS loss, $L_{TG}$ transfers the softened posterior of the frozen PASEM-AS teacher, $L_{MM}$ matches the fixed MM-KWS-style probability targets, $L_{rank}$ preserves their pairwise score ordering, and $L_{query}$ provides query-level contrastive regularization. The temperatures and loss coefficients are fixed across all reported Stage 4 runs [12,23].

Both teacher signals are frozen and used only during training. At inference, PASEM-TR uses only the trained Tibetan-PASEM student and the decision threshold defined by the evaluation protocol; neither teacher is loaded.

### 2.8. Operating-Point Selection and Metrics

For each configuration and random seed, the operating threshold τ* is selected on seen_cv by maximizing F1 subject to a false alarm rate (FAR) ≤ 0.10. Ties are resolved first by lower FAR and then by the higher threshold. The selected threshold is applied unchanged to the corresponding held-out evaluation conditions. Held-out labels, held-out score distributions, and test-specific thresholds are not used for operating-point selection [10,12,15].

$$\tau^{*}=\arg\underset{\tau }{\max}F1_{cv}\left(\tau \right),\;subject\;to\;FAR_{cv}\left(\tau \right)\leq 0.10.$$

The complete stage-wise and query-view analyses use the original pair index. For the corrected-index re-evaluation, $\tau^{*}$ is reselected independently for each random seed on cleaned seen_cv and is then applied unchanged to cleaned seen_test, unseen_test, and the confusable-negative condition. For the FAR–FRR visualization reported in Section 3.4, the aligned seed-2028 score archives of PASEM-AS, PASEM-TR, and G-only/Direct-ST are evaluated over the same 27,577 cleaned seen_cv pairs and labels. The curves characterize the false alarm rate–false rejection rate (FAR–FRR) trade-off, and the marked points correspond to the thresholds selected by the same FAR-constrained validation rule. Held-out scores and labels are not used to construct the curves or select the marked operating points.

The reported thresholded metrics are Accuracy, Precision, Recall, F1, FAR, and FRR. Accuracy measures the overall pair-level decision rate. Precision measures the proportion of predicted triggers that are correct, whereas Recall measures the proportion of positive pairs detected by the system. F1 summarizes the balance between Precision and Recall. FAR measures false triggers among negative pairs, and FRR measures missed triggers among positive pairs. The area under the receiver operating characteristic curve (AUC) and equal error rate (EER) are additionally reported when aligned pair-level score archives are available; these two metrics summarize score-level discrimination across thresholds, whereas the remaining metrics describe performance at the validation-selected operating point.

Three-seed results are reported as the mean and sample standard deviation across seeds 2026, 2027, and 2028. Seed-level 95% confidence intervals for the principal unseen_test results are calculated as:

$$\bar{x}\pm t_{0.975,2}\frac{s}{\sqrt{n}}$$

where $\bar{x}$ is the seed-level mean, s is the sample standard deviation, n = 3, and t_0.975,2_ = 4.3027. These intervals characterize variation across the reported training seeds and should not be interpreted as pair-level binomial confidence intervals.

The split roles are fixed before evaluation. The seen_train split is used for model fitting, and seen_cv or cleaned seen_cv is used for model selection and operating-threshold selection under the corresponding protocol. The held-out conditions are used only for final metric computation. Test labels, held-out score distributions, and test-specific threshold searches are not used for model selection, query-view selection, teacher-signal construction, or operating-point selection.

### 2.9. Implementation Details and Protocol-Level Reproducibility

The experiments were developed within the open-source WeKWS keyword-spotting framework [33]. WeKWS provides feature-extraction, checkpointing, and evaluation utilities, while the reported Tibetan-PASEM experiments add the structured written-query representation, Querybank198 pair construction, latent-window evidence matching, local verification, teacher-guided training settings, and split-aware evaluation workflow.

The reported system uses 16 kHz mono speech, 80-dimensional log-Mel filterbank features, a 25 ms frame length, a 10 ms frame shift, and approximately 2.0 s acoustic windows with a 0.8 s shift. The formal CNN-Conformer instantiation contains approximately 4.68 million parameters. The local verifier retains the five highest-scoring windows and uses a 256–128–1 multilayer perceptron with a dropout rate of 0.1. Additional model and training settings are specified in Section 2.3, Section 2.4, Section 2.5, Section 2.6, Section 2.7 and Section 2.8.

The released protocol-and-evaluation package contains the Querybank198 query metadata, original and cleaned pair indexes, negative-construction records, same-query control manifests, complete-file and decoded-PCM hash audits, acoustic-identity removal records, speaker and session-proxy statistics, development-to-held-out overlap matrices, threshold-selection and metric scripts, archived Tibetan-PASEM pair-level scores, per-seed thresholds, and score-alignment records [24,25,26,27].

These materials support reconstruction of the Querybank198 protocol, development-to-held-out audio correction, and score-level fixed-threshold evaluation. The package does not include the complete Tibetan-PASEM training implementation and therefore does not claim end-to-end model-training reproducibility. The accompanying README specifies the package scope, directory structure, required source resources, path configuration, expected split counts, score-file formats, evaluation commands, and known reproduction boundaries.

## 3. Results

### 3.1. Developmental Evidence and Formal Evaluation Protocol

Querybank50 and Querybank100 provided developmental evidence for the final Querybank198 protocol. Querybank50 established the feasibility of phonological query evidence and retrieval–verification matching, whereas Querybank100 exposed scaling, threshold-stability, and held-out-query difficulties. Querybank198 is the formal protocol used for the main stage-wise, query-view, confusable-negative, and corrected-index analyses.

### 3.2. Querybank198 Split-Independence Audit

Querybank198 contains 198 written Tibetan query entries derived from TIBMD@MUC, XBMU-AMDO31, Tibetan Greetings, and NICT-Tib1 [24,25,26,27]. The original protocol was constructed at the indexed query–speech pair level and contained 214,644 pairs referencing 91,988 WAV paths. Because a referenced recording can be evaluated against multiple written queries, the pair count is not equivalent to the number of independent recordings.

The complete-file and decoded-PCM audit identified recording-content overlap across the original development and held-out roles. The correction retained seen_train and removed 1271 pairs from seen_cv, 183 from seen_test, 9 from unseen_test, and 53 from the confusable-negative condition. The revised evaluation therefore contains 213,128 indexed pairs. Table 4 reports the corrected pair, recording, and source-qualified speaker counts.

After correction, no canonical WAV path, complete-file SHA-256 group, decoded-PCM SHA-256 group, source-qualified speaker, or derived session proxy is shared between seen_train or cleaned seen_cv and any held-out evaluation condition. The four source corpora do not provide a common official session identifier; session independence is therefore reported using conservative speaker-scoped proxies derived from corpus path and recording-condition metadata.

The three held-out conditions retain shared held-out recordings and speakers. Their results characterize different query roles over correlated acoustic pools and are not treated as statistically independent dataset replications. Table S1 reports the corrected split statistics, development-to-held-out overlap audits, and held-out-to-held-out recording and speaker reuse.

To make the split design transparent, Table 5 lists representative entries drawn from the Querybank198 query lists and phonology metadata. Seen entries evaluate whether training-exposed query forms remain detectable under held-out speech. Unseen entries evaluate held-out written-query transfer for query forms not used during model training. Confusable-negative entries are training-exposed query forms evaluated against targeted negative examples that share written components, syllables, approximate phonological cues, or locally overlapping acoustic evidence. Approximate cues are query-side metadata derived from inherited entries and rule-based fallback mappings [24,25,26,27,28,29].

### 3.3. Controlled Ablation Instantiations on Querybank198

Table 6 summarizes the complete Stage 1–Stage 4 ablation chain under the original pair-level Querybank198 protocol. The primary Stage 3/PASEM-AS system is independently evaluated under the corrected development-to-held-out audio-disjoint evaluation in Section 3.4. This separation preserves the complete stage-wise comparison while making the protocol used by each result explicit.

**Table 6:** Stage-wise results under the original Querybank198 pair index. Values are percentages; three-seed entries are reported as mean ± standard deviation.

Stage	Ablation Role	Split	AUC (%)	EER (%)	Recall (%)	F1 (%)
Stage 1	Candidate retrieval	seen_test	87.03	21.21	56.11	60.28
Stage 1	Candidate retrieval	unseen_test	77.68	29.49	31.92	36.92
Stage 1	Candidate retrieval	confusable-negative condition	89.46	18.67	61.39	67.52
Stage 2	Local verification	seen_test	87.75	20.27	59.19	61.70
Stage 2	Local verification	unseen_test	76.28	29.73	32.15	36.52
Stage 2	Local verification	confusable-negative condition	90.31	18.05	62.50	67.44
Stage 3	Acoustic strengthening	seen_test	89.49 ± 0.79	17.86 ± 1.08	60.76 ± 2.70	65.95 ± 2.38
Stage 3	Acoustic strengthening	unseen_test	72.51 ± 2.88	33.19 ± 2.03	18.48 ± 2.98	25.80 ± 2.71
Stage 3	Acoustic strengthening	confusable-negative condition	91.65 ± 0.48	15.49 ± 0.80	66.97 ± 1.93	71.43 ± 2.19
Stage 4	Transfer regularization	seen_test	89.65 ± 0.02	18.31 ± 0.06	67.46 ± 1.77	65.27 ± 0.14
Stage 4	Transfer regularization	unseen_test	77.36 ± 0.21	29.93 ± 0.00	33.85 ± 8.22	35.25 ± 4.17
Stage 4	Transfer regularization	confusable-negative condition	92.20 ± 0.06	15.53 ± 0.14	72.56 ± 1.43	70.77 ± 0.28

Note: The legacy hardest_test file is reported as the confusable-negative condition. Corrected-index PASEM-AS results are reported in Table 7; the G-only and PASEM-TR seed-2028 corrected-index sensitivity checks are reported in Table S1d.

**Table 7:** Three-seed corrected-index re-evaluation of PASEM-AS. Thresholds were reselected independently on cleaned seen_cv and applied unchanged to the three held-out conditions. Values are mean ± standard deviation across seeds 2026–2028.

Split	Threshold	Accuracy (%)	Precision (%)	Recall (%)	F1 (%)	FAR (%)	FRR (%)
seen_test	0.757 ± 0.038	89.50 ± 0.70	71.67 ± 2.92	61.50 ± 2.54	66.16 ± 2.14	4.89 ± 0.71	38.50 ± 2.54
unseen_test	0.757 ± 0.038	82.09 ± 0.80	42.90 ± 3.17	19.51 ± 4.14	26.51 ± 3.42	5.34 ± 1.78	80.49 ± 4.14
confusable-negative	0.757 ± 0.038	91.20 ± 0.54	76.63 ± 2.64	68.11 ± 1.22	72.10 ± 1.39	4.17 ± 0.62	31.89 ± 1.22

The AUC and EER summarize score ordering across all thresholds, whereas Recall is measured at the seed-specific threshold selected on seen_cv. Small AUC/EER variation and larger unseen_test Recall variation are therefore compatible when positive scores are concentrated near the selected operating boundary.

Under the original pair-level protocol, the controlled instantiations exhibit different condition-specific operating profiles. PASEM-AS/Stage 3 provides the highest seen_test and confusable-negative F1, whereas PASEM-TR/Stage 4 provides the more favorable unseen-query transfer profile. On unseen_test, Stage 4 increased Recall from 18.48 ± 2.98% to 33.85 ± 8.22% and F1 from 25.80 ± 2.71% to 35.25 ± 4.17% relative to Stage 3. The Stage 4 seed-level 95% confidence intervals were 13.43–54.27% for Recall and 24.89–45.61% for F1, indicating substantial variability across the three formal seeds. The Stage 4 result is therefore interpreted as partial and seed-sensitive transfer to held-out written queries. Because the three evaluation conditions differ in query exposure and negative construction, their split-level F1 values are not interpreted as an ordered difficulty scale. The larger unseen_test Recall variation is consistent with the thresholded operating-point sensitivity described above and with the limited positive support detailed in Section 3.4.

### 3.4. Fixed-Threshold and FAR–FRR Operating-Point Analysis

Table 6 evaluates the complete Stage 1–4 ablation chain, whereas Table 7 re-evaluates the primary PASEM-AS operating profile after development-to-held-out audio correction. The archived model checkpoints are retained, and only pair alignment and validation-based threshold selection are repeated on the cleaned indexes.

In the corrected-index sensitivity analysis, Stage 3/PASEM-AS achieved a 66.16 ± 2.14 F1 with a 4.89 ± 0.71 FAR on seen_test, a 26.51 ± 3.42 F1 with a 5.34 ± 1.78 FAR on unseen_test, and a 72.10 ± 1.39 F1 with a 4.17 ± 0.62 FAR on the confusable-negative condition. Relative to the original pair-level evaluation, the mean F1 changed by +0.12 percentage points on seen_test, +0.52 points on unseen_test, and +0.01 points on the confusable-negative condition. These small changes indicate that the fixed-model Stage 3 score profile is stable after development-to-held-out recording-content correction.

The confusable-negative row contains 20 training-exposed query forms with above-average positive-pair coverage in seen_train, whereas seen_test contains the broader seen-query inventory. The difference between their aggregate F1 values therefore combines query-set composition and negative-set construction. Section 3.5 separates these factors by evaluating the same 20 query forms and identical positive pairs against standard seen_test negatives and targeted confusable negatives under the same validation-selected thresholds.

For the corrected unseen_test condition, the seed-level 95% confidence intervals were 9.23–29.79% for Recall and 18.01–35.01% for F1. This condition contains 79 query forms, 2697 indexed pairs, and 451 positive pairs, corresponding to 5.71 positive pairs per query on average. The limited positive support per query and the use of three formal training seeds constrain the precision of the aggregate unseen estimate. Accordingly, unseen_test is interpreted as a held-out written-query transfer diagnostic and is reported separately from familiar-query detection.

**Figure 3:**
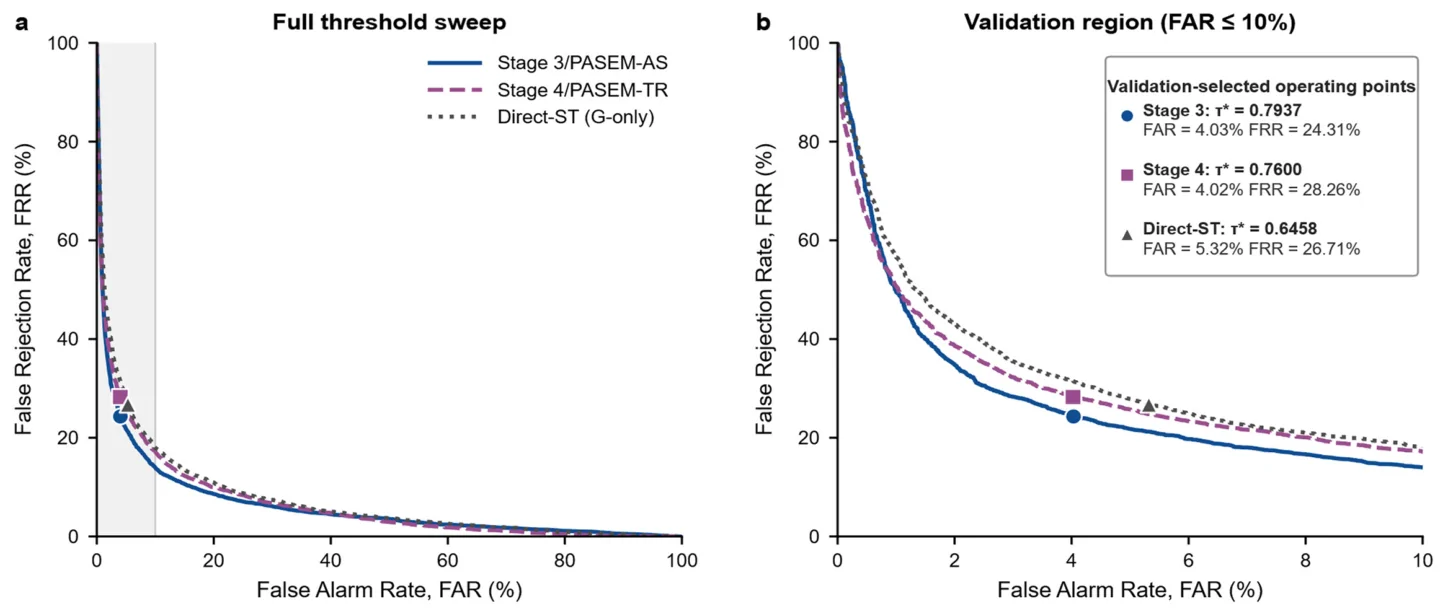
FAR–FRR characteristics on cleaned seen_cv using aligned seed-2028 scores. (**a**) Full threshold sweep. (**b**) FAR ≤ 10% region; markers indicate the validation-selected operating points for PASEM-AS, PASEM-TR, and G-only/Direct-ST. FAR, false alarm rate; FRR, false rejection rate.

Figure 3 complements the three-seed Stage 3 summary in Table 7 with a pair-aligned seed-2028 comparison on cleaned seen_cv. At the validation-selected operating points, Stage 3/PASEM-AS used τ* = 0.7937 and achieved FAR = 4.03% with FRR = 24.31%. Stage 4/PASEM-TR used τ* = 0.7600 and achieved FAR = 4.02% with FRR = 28.26%, whereas Direct-ST (G-only) used τ* = 0.6458 and achieved FAR = 5.32% with FRR = 26.71%. Stage 3 therefore produced the lowest FRR among the three validation-selected operating points while maintaining the FAR at approximately 4%. Across the complete threshold sweep, the three systems exhibit distinct FAR–FRR trade-offs that place the selected fixed operating points within their broader score-level behavior.

### 3.5. Same-Query Control and Query-Level Analysis of the Confusable-Negative Condition

To separate query exposure from negative-set composition, the same 20 training-exposed query forms and the same 2081 positive pairs were evaluated with two negative sets: ordinary negatives drawn from cleaned seen_test and targeted negatives from the confusable-negative condition. The threshold selected independently for each PASEM-AS seed on cleaned seen_cv was applied unchanged to both conditions.

Across seeds 2026–2028, ordinary negatives produced a 71.89 ± 1.71% F1 and a 4.29 ± 0.75% FAR, whereas targeted negatives produced a 72.10 ± 1.39% F1 and a 4.17 ± 0.62% FAR. The deterministic count-matched analysis yielded the same pattern. The targeted construction therefore does not define a universally harder test condition at the aggregate level.

**Figure 4:**
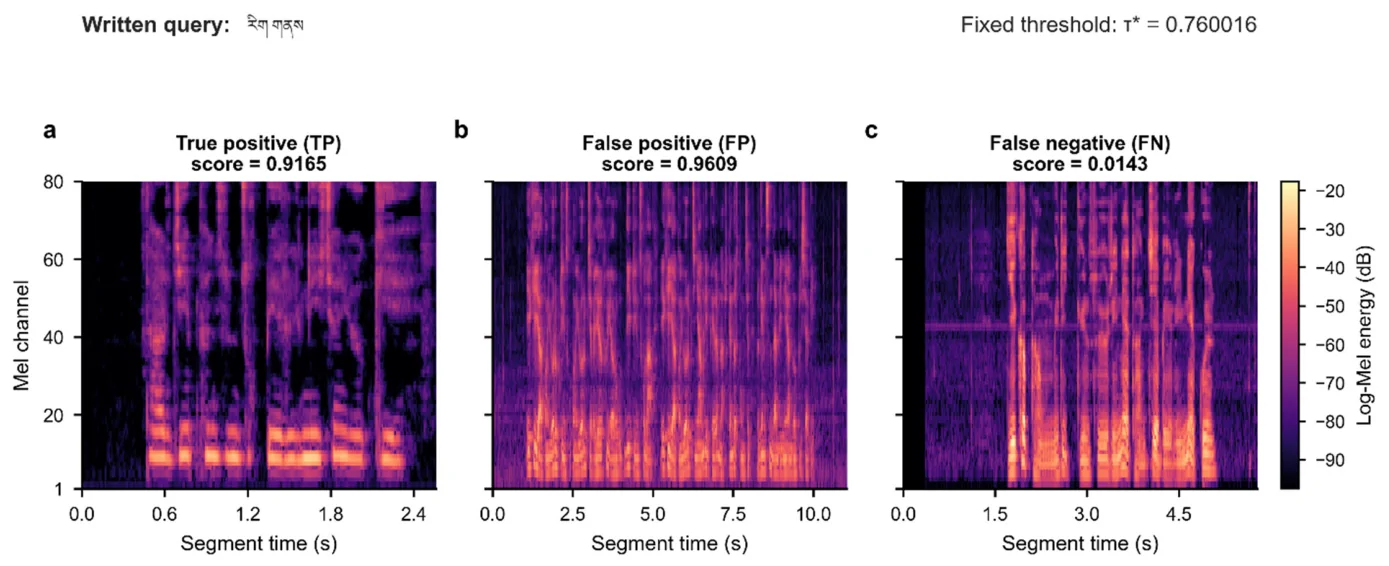
Representative pre-normalization 80-channel log-Mel spectrograms for the written query རིག གནས under the fixed PASEM-TR operating point: (**a**) true positive, score = 0.9165; (**b**) false positive, score = 0.9609; and (**c**) false negative, score = 0.0143. The fixed threshold is τ* = 0.760016. Each panel shows the full evaluated speech segment from which candidate windows were generated, using the same 16 kHz, 80-channel log-Mel configuration and shared color scale. Panel durations follow the corresponding evaluated speech segments. The Tibetan-script query label is retained because it denotes the exact written-query input used in this evaluation.

**Figure 5:**
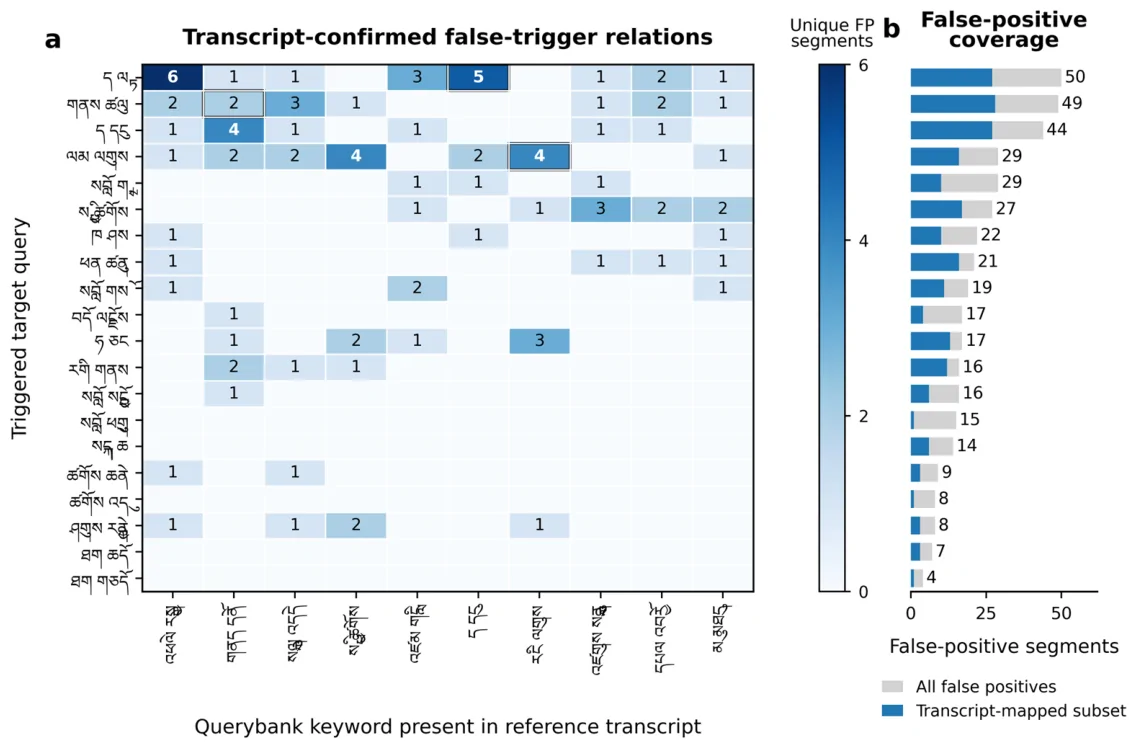
Transcript-confirmed false-trigger relations for PASEM-TR seed 2028 on the cleaned confusable-negative condition at the fixed threshold τ* = 0.760016 selected on cleaned seen_cv. (**a**) Rows denote the 20 triggered target queries, and columns show the 10 Querybank198 written forms most frequently present in the reference transcripts of false-positive speech segments. Cell values report unique false-positive segment counts; outlined nonzero cells indicate predefined phonological-neighbor relations. (**b**) Total false-positive counts and the transcript-mapped subset for the same target queries. A speech segment may contribute to multiple relation cells when several Querybank forms occur in its transcript. The visualization is a transcript-confirmed relation map rather than a unique-label multiclass confusion matrix. Tibetan-script row and column labels are retained because they denote the exact Querybank198 written-query forms used in the false-trigger analysis; they function as query identifiers rather than explanatory prose.

Its value is diagnostic. The Stage 4/PASEM-TR seed-2028 analysis applies one validation-selected threshold to all 12,475 cleaned confusable-negative pairs and characterizes how false alarms and missed targets vary across individual queries. Table 8 summarizes representative query-level cases, and Figure 4 and Figure 5 visualize acoustic decisions and recurrent transcript-confirmed false-trigger relations.

Table 8 reports representative higher-error and stable cases.

The confusable-negative condition contains both higher-error and stable query cases. Tibetan-script query forms are retained in this subsection as the exact written inputs used for evaluation; the surrounding English text provides their diagnostic interpretation. Among the representative queries in Table 8, རིག གནས and གནས ཚུལ show the highest query-level FAR values, at 8.00% and 7.99%, respectively. Several queries, including ཐག གཅོད, ཧ ཅང, སྤྱི ཚོགས, and སློབ གྲྭ, maintain substantially lower FARs under the same global threshold. None of the 20 queries has zero recall. These results reveal heterogeneous query-level false-trigger and missed-trigger behavior that is not captured by the aggregate ordinary-versus-targeted comparison alone.

Figure 4 provides a deterministic segment-level acoustic comparison for the written query རིག གནས under the same Stage 4/PASEM-TR operating point. The query was selected using the predefined query-level FAR criterion among queries containing at least one true positive, one false positive, and one false negative. Within this query, the true-positive example was selected nearest to the median true-positive score, the false-positive example was the highest-scoring negative pair, and the false-negative example was the lowest-scoring positive pair. Each panel shows the full evaluated speech segment from which the candidate acoustic windows were generated. All panels use the acoustic preprocessing defined in Section 2.4 and a shared display scale.

At the fixed threshold, the false-positive segment received a posterior of 0.9609, exceeding both the decision threshold and the representative true-positive score of 0.9165, whereas the false-negative target received a posterior of 0.0143. The shared display scale provides segment-level acoustic context for these contrasting decisions under the same written query. These examples complement the query-level metrics in Table 8, while recurrent false-trigger relations are examined in Figure 5.

To identify recurrent query relations within the false-positive set, each Stage 4/PASEM-TR seed-2028 false-positive pair was linked to its reference transcript through the archived pair key. After Tibetan text normalization, all 198 Querybank written forms were matched as exact contiguous syllable-token sequences, excluding the triggered target query itself. Figure 5a reports, for each of the 20 target queries, the number of unique false-positive speech segments containing each of the 10 most frequent transcript-present Querybank forms. Figure 5b compares the transcript-mapped subset with all false positives for the same target queries. Because a reference transcript may contain more than one Querybank form, a single segment can contribute to multiple cells; the map records transcript-confirmed relations without assigning a unique competing keyword to each false positive.

Among the 421 false-positive pairs, 215 contained at least one other Querybank written form under the exact token-matching rule. The displayed relation matrix was sparse: the largest cell contained six unique false-positive speech segments, while many target–keyword combinations were absent. Several recurrent cells coincided with predefined phonological-neighbor relations, whereas others did not. The relation map therefore localizes repeated query-specific false-trigger patterns. Table S3 reports the aggregate and representative query-level results, the same-query ordinary-negative controls, and the transcript-confirmed coverage summary, while File S1 provides the complete 20-query machine-readable metrics, the full 20 × 198 relation matrix, long-format relation records, and per-query coverage totals.

### 3.6. Query-View Sensitivity and Grapheme-Only Control

We compare four Stage 3 query-evidence settings—G-only, P-only, G + P, and G + P + D—using the same Querybank198 split files, acoustic encoder, training schedule, and seen_cv operating-point policy. G-only also serves as the Direct-ST internal control. The comparison therefore isolates query-side evidence behavior without changing the acoustic model or applying test-specific threshold tuning. Table 9 summarizes this controlled query-view comparison.

The query-view analysis reveals a split-dependent evidence trade-off. G-only/Direct-ST provides the highest seen_test and confusable-negative F1 scores, showing that normalized Tibetan graphemic evidence is a strong familiar-query anchor. P-only provides the highest unseen_test F1 but also the highest unseen_test FAR, indicating that approximate phonological cues carry transfer-relevant information together with increased false-trigger sensitivity.

The G + P result indicates a non-additive interaction between graphemic and approximate phonological evidence under the shared matcher. The full G + P + D representation provides the most conservative FAR on seen_test and the confusable-negative condition while retaining a competitive F1. Tibetan-PASEM is therefore interpreted as a jointly learned evidence-matching framework in which the three query views contribute differently to familiar-query discrimination, held-out-query transfer, and false-alarm control [9,10,12,15].

### 3.7. Fixed-Budget Reference Implementations Under Declared Input Assumptions

MM-KWS, PhonMatchNet, and Metric-UD-KWS originate from different user-defined keyword-spotting settings and are therefore reported as fixed-budget reference implementations rather than as convergence-equivalent baselines [6,9,12]. MM-KWS-style and PhonMatchNet provide written- or phonology-guided reference points under Querybank198, whereas Metric-UD-KWS retains its spoken-enrollment query-by-example assumption and is reported as a separate diagnostic reference. Table 10 summarizes the query assumptions, fitting data, selection data, and evaluation roles used in this study.

The MM-KWS-style implementation retains the multimodal prompt-fusion motivation of MM-KWS while adapting its query and speech inputs to Querybank198 [12]. The original language-specific query pipeline is replaced with Tibetan graphemic and approximate phonological token streams. The Querybank198 implementation uses a task-specific acoustic encoder and transformer-based fusion layers, while the original support-speech and AudioLM pathways are omitted. Model parameters are fitted on seen_train for 10 epochs using AdamW, a learning rate of 2 × 10^−4^, a weight decay of 1 × 10^−4^, a batch size of 8, gradient accumulation over two batches, a negative-to-positive sampling ratio of 3:1, and an auxiliary-loss weight of 0.35. Checkpoint selection is restricted to seen_cv.

The PhonMatchNet reference retains the phonology-guided matching core of the original method and adapts the query interface to Querybank198 [9]. The original English G2P and Google speech-embedding pathways are replaced with Tibetan pseudo-phonological token sequences. The Tibetan dataset adapter, tokenization, pair-level evaluator, and FAR-constrained threshold procedure were implemented for the present protocol. Model fitting uses seen_train, checkpoint selection is restricted to seen_cv, and held-out audio is not used as query enrollment. The formal launcher uses Adam with a learning rate of 1 × 10^−4^, zero weight decay, a batch size of 4, a 10-epoch budget, and random seed 42. The checkpoint filename is best.pt, and selection follows the best seen_cv F1 rule. These settings and the corresponding archive-status disclosure are reported in Table S2 and File S1; the executed epoch history, selected epoch number, checkpoint binary, and raw score archive are not included in the available source snapshot.

Metric-UD-KWS is retained as an enrollment-based fair query-by-example diagnostic rather than as a written-query reference [6]. Its ResNet15 embedding network is trained with an angular prototypical objective using positive seen_train speech examples. Five spoken examples are used to construct each query prototype. For training-exposed queries, enrollment examples are selected from seen_train positives. For query forms absent from seen_train, split-local positive utterances are used for enrollment; the selected enrollment trials and trials sharing the same audio are excluded before scoring. Metric-UD-KWS therefore uses a different query assumption from Tibetan-PASEM, MM-KWS-style, and PhonMatchNet.

The three reference implementations were evaluated under archived method-specific fixed budgets. Because their architectures, query assumptions, and recommended training schedules differ, convergence-equivalent optimization and equal tuning effort are not claimed. Table S2 reports the retained and reimplemented components, formal launcher settings, training budgets, query assumptions, and selection rules. File S1 provides the corresponding configuration files, command records, checkpoint filenames, archive-status disclosures, and score-generation audit summaries. Executed epoch-level histories, selected epoch numbers, checkpoint binaries, and raw baseline score archives are not part of the released package.

Table 11 reports the corresponding fixed-threshold results. MM-KWS-style and PhonMatchNet are interpreted as written- or phonology-guided protocol references, whereas Metric-UD-KWS is an enrollment-based diagnostic with a different query assumption. The values provide Querybank198 context and are not interpreted as a convergence-equivalent ranking of the original systems.

Relative to the two fixed-budget written- or phonology-guided reference implementations, Stage 3/PASEM-AS provides higher seen_test and confusable-negative F1 scores than MM-KWS-style and PhonMatchNet. These differences contextualize the Stage 3 operating profile under Querybank198; they are not interpreted as an unrestricted ranking of the original methods or as evidence of convergence-equivalent optimization.

MM-KWS-style has two roles in this study. It is reported as a fixed-budget protocol reference, and its archived pair-level probabilities provide one of the two training-time teacher signals for Stage 4/PASEM-TR. On unseen_test, MM-KWS-style achieved a 36.19% F1, compared with 35.25 ± 4.17% for Stage 4/PASEM-TR. Stage 4 versus MM-KWS-style is therefore treated as a teacher–student diagnostic rather than as an independent head-to-head comparison. The Stage 4 experiment evaluates how fixed external score supervision changes the held-out-query transfer behavior of the Tibetan-PASEM student.

Metric-UD-KWS is reported as an enrollment-based fair-QBE diagnostic and is not treated as a direct written-query comparator. The principal evidence for Tibetan-PASEM is provided by the controlled Stage 1–4 instantiations, query-view analysis, fixed-threshold operating-point evaluation, same-query negative control, and development-to-held-out audio-disjoint sensitivity analysis. The external implementations provide complementary protocol-level context.

## 4. Discussion

### 4.1. Main Findings and Method Contribution

Tibetan-PASEM links structured Tibetan query evidence with local acoustic-window evidence through latent-window aggregation and top-ranked local verification. Under the corrected evaluation, PASEM-AS achieved a 66.16 ± 2.14% F1 on seen_test and 72.10 ± 1.39% on the confusable-negative condition, while unseen_test remained lower at 26.51 ± 3.42%. In the complementary stage-wise analysis on the original pair index, PASEM-TR improved the held-out-query operating profile relative to PASEM-AS, although the gain varied across seeds. These results support separate reporting of familiar-query detection, held-out written-query transfer, and query-specific false-trigger behavior.

Under the original pair index, PASEM-TR increased the unseen_test F1 from 25.80 ± 2.71% to 35.25 ± 4.17% and the Recall from 18.48 ± 2.98% to 33.85 ± 8.22%. The corresponding seed-level confidence intervals indicate substantial run-to-run variation. The present evidence therefore supports partial held-out-query transfer, while broader query coverage and lower transfer variance define the next evaluation target.

### 4.2. Query-Evidence Trade-Offs and Held-Out Transfer

The query-view analysis shows a split-dependent evidence trade-off. Graphemic evidence provides the strongest familiar-query anchor, whereas phonology-only evidence gives the highest unseen_test F1 together with the highest unseen_test FAR. The full representation provides a more conservative FAR profile on seen_test and the confusable-negative condition while retaining a competitive F1. Approximate phonological and dialect-related information therefore act as complementary evidence sources that shape transfer and false-alarm behavior [9,10,12,15,24,25,26,27,28,29].

PASEM-TR increased the unseen_test F1 from 25.80 ± 2.71% to 35.25 ± 4.17% and the Recall from 18.48 ± 2.98% to 33.85 ± 8.22%. The corrected unseen_test contains 451 positive pairs across 79 query forms, and the seed-level confidence intervals indicate substantial run-to-run variation. The present evidence therefore supports partial held-out-query transfer, while broader query coverage and lower transfer variance define the next evaluation target.

### 4.3. Confusable-Negative Diagnosis

The 20 confusable-negative query forms are training-exposed and have above-average positive-pair coverage. The same-query control therefore holds the query inventory and positive pairs fixed while changing the negative construction. Ordinary and targeted negatives produced similar aggregate FAR values of 4.29 ± 0.75% and 4.17 ± 0.62%, respectively. The targeted construction is therefore interpreted as a condition-specific diagnostic rather than as a universally harder test split.

Its diagnostic value appears at the query level. Table 8 and Figure 4 and Figure 5 show heterogeneous false-trigger and missed-target behavior under one global threshold, including recurrent transcript-confirmed relations and stable query cases that are averaged out by the aggregate comparison.

### 4.4. Split Integrity and Operating-Point Evaluation

The split audit separates pair-level KWS evaluation from recording-level independence control. Complete-file and decoded-PCM hashing, source-qualified speaker identifiers, and speaker-scoped session proxies produced a corrected 213,128-pair protocol with no identified development-to-held-out overlap under the reported criteria. At fixed PASEM-AS checkpoints, the corrected-index F1 changed by +0.12, +0.52, and +0.01 percentage points on seen_test, unseen_test, and the confusable-negative condition, respectively.

The three held-out conditions retain partial held-out-to-held-out recording and speaker reuse. They are therefore interpreted as correlated condition-specific evaluations rather than independent dataset replications. Figure 3 further shows that PASEM-AS, PASEM-TR, and G-only/Direct-ST occupy different FAR–FRR trade-offs even when their selected operating points lie in a similar validation FAR region.

### 4.5. Comparison Scope and Deployment Extensions

MM-KWS-style and PhonMatchNet are fixed-budget references adapted to the written-query protocol, whereas Metric-UD-KWS retains spoken enrollment and is reported as a fair query-by-example diagnostic. Their results provide Querybank198 context under declared input assumptions rather than convergence-equivalent rankings of the original methods. Because MM-KWS-style probabilities are used during PASEM-TR training, the PASEM-TR versus MM-KWS-style result is interpreted as a teacher–student diagnostic.

The present experiments evaluate pre-constructed query–speech pairs and approximately 2.0 s local windows. Continuous-stream evaluation requires long-form audio, trigger-merging rules, a fixed hardware target, and metrics such as false alarms per hour, missed events per hour, latency, real-time factor, memory use, and noise/channel robustness. These constitute the next deployment-oriented evaluation stage.

## 5. Conclusions

This study presented Tibetan-PASEM, a phonology-aware speech evidence-matching method for segment-level Tibetan written-query keyword spotting. Querybank198 was audited at the query-pair, recording-content, source-qualified speaker, and session-proxy levels, producing a corrected 213,128-pair evaluation with no identified development-to-held-out overlap under the reported criteria. In the corrected three-seed re-evaluation, PASEM-AS achieved 66.16 ± 2.14% F1 on seen_test, 26.51 ± 3.42% on unseen_test, and 72.10 ± 1.39% on the confusable-negative condition.

Under the original pair-index stage-wise analysis, PASEM-TR improved held-out-query Recall and F1 relative to PASEM-AS, while the seed-level confidence intervals characterize the variability of this transfer behavior. Together, the results establish strong familiar-query discrimination, an audited fixed-threshold evaluation protocol, and query-level false-trigger diagnostics. Future evaluation will extend held-out-query coverage and assess continuous-stream operation under false alarms per hour, latency, and resource constraints.

## Figures and Tables

**Table 1:** Notation and main components used in Tibetan Phonology-Aware Speech Evidence Matching (Tibetan-PASEM).

Symbol	Meaning in This Paper
q	Tibetan written keyword query
x	Utterance or candidate acoustic window
y	Pair-level binary keyword spotting (KWS) label
g(q)	Normalized graphemic view of the Tibetan query
p(q)	Approximate phonological view of the Tibetan query
d(q)	Dialect-related syllable/pronunciation feature vector
$e_{q}$	Query evidence embedding
$h_{i}$	Acoustic-window evidence embedding for the i−th window
$l_{i}$	Window-level matching logit
W(x)	Candidate-window set extracted from an utterance
τ	Validation-selected decision threshold
ŷ	Thresholded KWS decision
Z	Latent acoustic-window variable
$\pi_{i}\left(q,x\right)$	Local query–window match probability
sret (q,x)	Normalized log-sum-exp matcher score
u(q,x)	Top-five local evidence vector used by the verifier
sθ (q,x)	Local-verifier output logit
$P_{\theta }\left(Y=1|q,x\right)$	Final Tibetan-PASEM posterior

**Table 2:** Materials released for Querybank198 protocol reconstruction, audit, and score-level evaluation.

Component	Released Item	Supported Reproduction Role
Query metadata	198-query list, written forms, query IDs, phonology-source categories, and syllable-level features	Reconstructs the written-query side of the protocol
Split definitions	Original and cleaned seen_train, seen_cv, seen_test, unseen_test, and legacy hardest_test/confusable-negative pair files	Fixes the training, validation, and held-out evaluation roles
Pair indexes	Pair keys, query IDs, labels, source-resource IDs, utterance IDs, and configurable path-mapping keys	Reconstructs the indexed query–speech evaluation
Negative construction and same-query control	Negative-sampling rules, confusable-negative annotations, ordinary-negative control manifests, and count-matched records	Reconstructs standard, targeted, and same-query negative-composition evaluations
Recording-content audit	Canonical paths, complete-file SHA-256, decoded-PCM SHA-256, acoustic-identity groups, and removed-pair manifests	Reconstructs the development-to-held-out audio correction
Speaker/session audit	Source-qualified speaker identifiers and speaker-scoped session-proxy overlap matrices	Audits speaker and proxy-session independence
Evaluation scripts	Cleaned seen_cv threshold search, fixed-threshold testing, and metric computation	Recalculates Accuracy, Precision, Recall, F1, FAR, FRR, AUC, and EER
Evaluation configurations and score records	Evaluation configuration records, archived Tibetan-PASEM pair-level scores, thresholds, and score-alignment records	Audits the reported operating points and reproduces score-level metric recalculation; does not reproduce model training

**Table 3:** Source categories for approximate phonological representations in Querybank198.

Phonology Source Category	Queries	Interpretation
inherited_exact_old_phonology	18	Exact entry inherited from earlier phonology resources
inherited_syllable_old_phonology	3	Syllable-level inherited entry
hybrid_old_syllable_plus_rule_approx	42	Old syllable mapping plus rule-based fallback
rule_based_wylie_approx	135	Rule-based Wylie-style approximation

**Table 4:** Querybank198 statistics after development-to-held-out audio-disjoint correction.

Split	Queries	Pairs	Positive	Negative	Unique Recordings	Unique Speakers	Role
seen_train	99	127,536	31,884	95,652	63,033	1647	Model training
seen_cv	99	27,577	4590	22,987	10,891	381	Validation and threshold selection
seen_test	99	42,843	7151	35,692	16,549	397	Seen-query evaluation
unseen_test	79	2697	451	2246	2498	81	Held-out written-query transfer
confusable-negative	20	12,475	2081	10,394	9155	219	Confusable-negative evaluation

Note: “Unique recordings” denotes canonical referenced WAV paths after the development-to-held-out correction. Speaker counts use source-qualified speaker identifiers. Counts are reported separately for each condition and are not additive across the held-out conditions because those conditions partially reuse held-out acoustic material. The released pair file for the confusable-negative condition retains the legacy filename hardest_test_pairs.jsonl.

**Table 5:** Representative Tibetan queries in Querybank198.

Tibetan Query	Role	Syllables	Phonology Source	Evaluation Function
ཁས ལེན	seen	2	hybrid_old_syllable_plus_rule_approx	Held-out detection of a training-exposed query form
སྤྱི ཚོགས	seen	2	hybrid_old_syllable_plus_rule_approx	Seen-query discrimination under source variation
ཆབ སྲིད	seen	2	rule_based_wylie_approx	Written-query matching for a training-exposed form
ཚེས བཅུ	unseen	2	rule_based_wylie_approx	Held-out written-query transfer evaluation
རིམས ནད	unseen	2	rule_based_wylie_approx	Held-out transfer evaluation from approximate cues
གློག འཕྲིན	unseen	2	rule_based_wylie_approx	User-defined written-query transfer evaluation
གནས ཚུལ	confusable-negative	2	inherited_exact_old_phonology	Targeted confusable-negative evaluation
སྐད ཆ	confusable-negative	2	inherited_exact_old_phonology	Targeted confusable-negative evaluation
སློབ གྲྭ	confusable-negative	2	inherited_exact_old_phonology	Targeted confusable-negative evaluation
ཐག གཅོད	confusable-negative	2	rule_based_wylie_approx	Targeted confusable-negative evaluation

Note: Tibetan-script entries in the “Tibetan Query” column are retained because they are the exact written-query forms evaluated by the system; the adjacent English columns describe their protocol roles and evaluation functions.

**Table 8:** Representative query-level results for PASEM-TR seed 2028 on the cleaned confusable-negative condition. The global threshold was selected on cleaned seen_cv; pair counts are integers, and other values are percentages. Tibetan-script entries are retained as the exact written-query inputs evaluated by PASEM-TR; the English “Error Pattern” column provides the corresponding diagnostic interpretation.

Tibetan Query	Phonology Source	Positive	Negative	F1 (%)	FAR (%)	Recall (%)	Error Pattern
རིག གནས	hybrid_old_syllable_plus_rule_approx	40	200	42.25	8.00	37.50	elevated FAR and low recall
ཐག ཆོད	rule_based_wylie_approx	55	272	59.09	2.57	47.27	low recall
སྐད ཆ	inherited_exact_old_phonology	52	259	58.06	5.41	51.92	moderate discrimination
གནས ཚུལ	inherited_exact_old_phonology	123	613	72.12	7.99	78.86	elevated FAR
སྤྱི ཚོགས	hybrid_old_syllable_plus_rule_approx	234	1162	73.61	2.32	64.96	stable discrimination
སློབ གྲྭ	inherited_exact_old_phonology	187	937	82.93	3.10	81.82	stable discrimination
ཧ ཅང	rule_based_wylie_approx	260	1290	87.85	1.32	83.46	stable discrimination
ཐག གཅོད	rule_based_wylie_approx	66	330	88.00	1.21	83.33	stable discrimination

**Table 9:** Stage 3 query-view sensitivity under the original Querybank198 pair index. G-only also serves as the Direct-ST internal control. Values are percentages except for the validation threshold.

Variant	Query Evidence	seen_test F1 (%)	seen_test FAR (%)	unseen_test F1 (%)	unseen_test FAR (%)	Confusable-Negative Condition F1 (%)	Confusable-Negative Condition FAR (%)	CV Threshold
G-only/Direct-ST	grapheme_only	64.17	5.83	25.12	4.08	71.00	4.71	0.667194
P-only	phonology_only	54.80	9.04	33.67	12.86	61.58	7.80	0.423538
G + P	grapheme_phonology	63.11	5.67	23.72	6.70	69.22	5.39	0.772569
G + P + D	full	63.73	5.14	27.86	6.03	68.99	4.34	0.667259

**Table 10:** Query assumptions, data use, and evaluation roles of Tibetan-PASEM and the fixed-budget reference implementations. Detailed adaptation, formal launcher settings, training budgets, checkpoint-selection rules, archive-status disclosures, and score-generation audit summaries are provided in Table S2 and File S1.

Method	Query or Enrollment Input	Parameter Fitting	Model and Threshold Selection	Held-Out Audio Enrollment	Evaluation Role
Tibetan-PASEM	written query with graphemic, approximate phonological, and dialect-related evidence	seen_train	seen_cv	No	proposed written-query method
G-only/Direct-ST	normalized Tibetan written query	seen_train	seen_cv	No	internal grapheme-only control
MM-KWS-style	Tibetan graphemic and approximate phonological token streams; no support-speech branch	seen_train	seen_cv	No	fixed-budget multimodal-prompt reference
PhonMatchNet	Tibetan pseudo-phonological token sequence	seen_train	seen_cv	No	fixed-budget phonology-guided reference
Metric-UD-KWS fair-QBE	five spoken enrollment examples per query	seen_train positives	seen_cv	Yes for queries absent from seen_train; enrollment audio excluded from scoring	enrollment-based diagnostic reference

Note: “Fixed-budget” indicates that each implementation is evaluated using its archived method-specific training schedule and checkpoint-selection rule. Convergence-equivalent optimization and equal tuning effort are not claimed. Metric-UD-KWS uses spoken enrollment and is therefore not a same-interface written-query comparison. For its fair-QBE evaluation, split-local enrollment trials and trials sharing the enrollment audio are removed before scoring.

**Table 11:** Fixed-budget reference implementations under the original pair-level Querybank198 protocol and their declared query assumptions. MM-KWS-style and PhonMatchNet are written- or phonology-guided protocol references, whereas Metric-UD-KWS is an enrollment-based fair-QBE diagnostic. Values are percentages.

Method	Split	AUC (%)	EER (%)	Recall (%)	F1 (%)	FAR (%)
MM-KWS-style reference	seen_test	75.61	31.60	30.19	35.50	7.98
MM-KWS-style reference	unseen_test	80.45	24.55	28.60	36.19	5.90
MM-KWS-style reference	confusable-negative	76.37	30.69	29.31	35.75	6.93
PhonMatchNet fixed-budget reference	seen_test	67.91	36.17	23.68	26.49	11.03
PhonMatchNet fixed-budget reference	unseen_test	65.94	35.10	14.63	17.41	10.70
PhonMatchNet fixed-budget reference	confusable-negative	70.07	35.73	28.78	31.54	10.75
Metric-UD-KWS fair-QBE diagnostic	seen_test	55.67	46.12	13.11	15.86	10.44
Metric-UD-KWS fair-QBE diagnostic	unseen_test	64.27	40.81	27.32	18.37	14.82
Metric-UD-KWS fair-QBE diagnostic	confusable-negative	56.15	45.83	13.27	15.97	10.57

Note: Checkpoint or configuration selection and threshold selection are restricted to seen_cv. MM-KWS-style and PhonMatchNet use no held-out audio enrollment. Metric-UD-KWS uses five spoken enrollment examples per query; for query forms absent from seen_train, split-local positive enrollment utterances are used and excluded, together with trials sharing the same audio, before formal scoring. Effective launcher configurations, selection rules, archive-status disclosures, and score-generation audit summaries are provided in Table S2 and File S1.

## Data Availability

Querybank198 is released as a protocol-and-evaluation package rather than as redistributed speech audio. File S1 contains the query metadata, original and cleaned pair indexes, negative-construction and same-query control manifests, recording-content and split-overlap audits, configurable path mapping, validation-based threshold-selection and fixed-threshold evaluation scripts, archived Tibetan-PASEM pair-level scores, Figure 3, Figure 4 and Figure 5 source data, and effective configuration, checkpoint-selection-rule, archive-status, and score-generation-audit records for the fixed-budget reference implementations. The underlying speech recordings must be obtained from the original providers of TIBMD@MUC, XBMU-AMDO31, Tibetan Greetings, and NICT-Tib1 in accordance with their distribution terms [24,25,26,27]. The release supports protocol reconstruction, recording-content auditing, operating-point selection, score-to-pair alignment, and score-level metric recalculation. Complete end-to-end Tibetan-PASEM model-training code and raw baseline score archives are outside the scope of the current release.

## Supplementary Materials

The following supporting information can be downloaded at: https://www.mdpi.com/article/10.3390/s26165107/s1, Table S1, Querybank198 original and corrected split statistics, development-to-held-out recording-content, speaker, and session-proxy overlap audits, residual relationships among the cleaned held-out conditions, and corrected-index sensitivity results; Table S2, Tibetan-PASEM core implementation settings, Stage 4 teacher identities, temperatures and loss weights, and adaptation, formal launcher settings, training-budget, query-assumption, and selection-rule records for the fixed-budget reference implementations; Table S3, aggregate and representative query-level confusable-negative results, same-query ordinary-negative full-pool and count-matched controls, and transcript-confirmed false-trigger coverage summaries; File S1, Querybank198 protocol-and-evaluation package, including the README, path-mapping template, query metadata, original and cleaned pair indexes, audit manifests, complete 20-query machine-readable metrics, same-query control manifests, evaluation scripts, archived Tibetan-PASEM pair-level scores, Figure 3, Figure 4 and Figure 5 source data, effective reference configurations, command records, checkpoint-selection rules, archive-status disclosures, score-generation audit summaries, and SHA-256 manifest.

## Abbreviations

**Abbreviation**	**Meaning**
ASR	Automatic speech recognition
AUC	Area under the receiver operating characteristic curve
EER	Equal error rate
FAR	False alarm rate
FRR	False rejection rate
KWS	Keyword spotting
PASEM	Phonology-Aware Speech Evidence Matching
UD-KWS	User-defined keyword spotting
