# Supplementary material for: Tibetan-PASEM: Phonology-Aware Speech Evidence Matching for Low-Resource Tibetan Written-Query Keyword Spotting
Source: Sensors (Basel). 2026 Aug 12;26(16):5107. doi: 10.3390/s26165107 (PMC13517381; doi:10.3390/s26165107)
Supplement: Supplementary file 1 [file sensors-26-05107-s001.zip › Supplementary Information.pdf]

Supplementary Information for

Tibetan-PASEM: Phonology-Aware Speech Evidence Matching for Low-Resource Tibetan Written-Query Keyword Spotting

Yanze Guo, Xingmeng Guo, Zengguang Li, Jiaxin Song, Yuyang Gong, and Guanyu Li

Table S1. Querybank198 protocol correction, split audit, and corrected-index sensitivity results

Table S1(a). Original and corrected Querybank198 split statistics.

Split	Query role	Queries	Original pairs	Corrected pairs	Removed pairs	Positive pairs	Negative pairs	Unique recordings	Source-qualified speakers
seen_train	Model training	99	127,536	127,536	0	31,884	95,652	63,033	1,647
seen_cv	Validation and operating-point selection	99	28,848	27,577	1,271	4,590	22,987	10,891	381
seen_test	Training-exposed query evaluation	99	43,026	42,843	183	7,151	35,692	16,549	397
unseen_test	Held-out written-query transfer	79	2,706	2,697	9	451	2,246	2,498	81
confusable-negative	Targeted negative-composition diagnosis	20	12,528	12,475	53	2,081	10,394	9,155	219
Total	—	198 query entries	214,644	213,128	1,516	—	—	—	—

Note: "Unique recordings" denotes canonical referenced WAV paths after development-to-held-out correction. Speaker counts use source-qualified speaker identifiers. Counts are calculated separately for each condition and are not additive across the three held-out conditions because those conditions partially reuse held-out acoustic material. The released pair file for the confusable-negative condition may retain the legacy filename hardest_test_pairs.jsonl for compatibility. The corrected unseen_test contains 451 positive pairs for 79 query forms, corresponding to 5.71 positive pairs per query on average. The 20 confusable-negative queries account for 38,240 seen_train pairs, including 9,560 positive pairs, corresponding to 478 positive training pairs per query. The current manuscript reports these corrected pair, recording, and speaker statistics in Section 2.2 and Table 4

Table S1(b). Development-to-held-out overlap audit after Querybank198 correction

Development condition	Comparison condition	Shared canonical paths	Shared whole-file SHA-256 groups	Shared decoded-PCM SHA-256 groups	Shared source-qualified speakers	Shared session proxies
seen_train	cleaned seen_cv	0	0	0	0	0
seen_train	seen_test	0	0	0	0	0
seen_train	unseen_test	0	0	0	0	0
seen_train	confusable-negative	0	0	0	0	0
cleaned seen_cv	seen_test	0	0	0	0	0
cleaned seen_cv	unseen_test	0	0	0	0	0
cleaned seen_cv	confusable-negative	0	0	0	0	0

Note: Session proxies are conservative speaker-scoped identifiers derived from corpus path structure and recording-condition metadata. They are not official recording-session annotations supplied by the source corpora. No official session-independence claim is made beyond the metadata available from the four public speech resources. The audit supports zero identified recording-content, source-qualified speaker, and derived session-proxy overlap across the development-to-held-out boundary.

Table S1(c). Residual relationships among the cleaned held-out evaluation conditions

Held-out condition A	Held-out condition B	Shared referenced recordings	Shared source-qualified speakers	Interpretation
seen_test	unseen_test	2,254	78	Correlated held-out acoustic pools
seen_test	confusable-negative	8,371	201	Correlated held-out acoustic pools
unseen_test	confusable-negative	1,277	47	Correlated held-out acoustic pools

Note: These overlaps occur only among held-out evaluation conditions and do not cross the development-to-held-out boundary. The three held-out conditions are therefore interpreted as correlated condition-specific evaluations rather than as statistically independent test-set replications.

Table S1(d). Corrected-index sensitivity results

System	Seeds	seen_test F1 / FAR (%)	unseen_test F1 / FAR (%)	Confusable-negative F1 / FAR (%)	ΔF1: seen / unseen / confusable (percentage points)
PASEM-AS	2026–2028	66.16 ± 2.14 / 4.89 ± 0.71	26.51 ± 3.42 / 5.34 ± 1.78	72.10 ± 1.39 / 4.17 ± 0.62	+0.12 / +0.52 / +0.01
G-only/Direct-ST	2028	64.15 / 6.02	25.24 / 4.27	70.73 / 4.90	+0.02 / +0.36 / −0.27
PASEM-TR	2028	64.82 / 4.52	27.49 / 5.34	71.36 / 4.05	−0.08 / 0.00 / −0.08

Note: PASEM-AS values are mean ± sample standard deviation across the three formal seeds. G-only/Direct-ST and PASEM-TR are seed-2028 score-level sensitivity checks. For every system, the operating threshold was selected on cleaned seen_cv and applied unchanged to the three cleaned held-out conditions. The PASEM-TR row does not replace the three-seed PASEM-TR result reported under the original stage-wise protocol. The corrected-index changes are small, supporting the stability of the archived score profiles after development-to-held-out recording-content correction.

Table S2. Tibetan-PASEM settings, Stage 4 teacher configuration, and fixed-budget reference-implementation records

Table S2(a). Effective Tibetan-PASEM architecture and evaluation settings

Component	Effective setting
Task	Segment-level Tibetan written-query keyword spotting
Query input	Normalized graphemic, approximate phonological, and dialect-related evidence
Speech sampling rate	16 kHz, mono
Acoustic feature	80-dimensional log-Mel filterbank
Frame length / frame shift	25 ms / 10 ms
Candidate-window length	Approximately 2.0 s
Candidate-window shift	0.8 s
Acoustic encoder	CNN-Conformer
Acoustic-window pooling	Mean pooling
Pair-level matcher aggregation	Normalized log-sum-exp
Log-sum-exp parameter	(\gamma = 1.0)
Windows retained for local verification	Five highest-scoring acoustic windows
Verifier input	Selected acoustic-window embeddings, matching logits, and query-side feature vector
Local-verifier architecture	256–128–1 multilayer perceptron
Hidden-layer activation	SiLU
Dropout rate	0.1
Layer normalization	Applied after the first hidden layer

Component	Effective setting
Final posterior	Sigmoid of the local-verifier output logit
Approximate parameter count	4.68 million
Operating-point data	seen_cv or cleaned seen_cv, according to the reported protocol
Operating-point rule	Maximize F1 subject to FAR ≤ 0.10
Tie-breaking rule	Lower FAR, followed by the higher threshold
Held-out threshold tuning	None
Separate linear retrieval–verification posterior coefficient	Not used

Note: Matcher evidence enters the verifier through the selected window embeddings and their matching logits. The reported implementation does not use a separately tuned linear coefficient to combine two independent posterior probabilities. The local-verifier architecture, top-five rule, $\gamma = 1.0$*, dropout, and operating-point constraint are explicitly described in Sections 2.5–2.8.*

Table S2(b). Stage 4/PASEM-TR teacher signals and loss configuration

Item	Teacher signal 1	Teacher signal 2
Identity	Frozen Stage 3/PASEM-AS model	Archived MM-KWS-style pair-level probabilities
Role	Internal teacher posterior	External score-level teacher target
Architecture/source	Tibetan-PASEM query encoder, CNN-Conformer acoustic encoder, query–window matcher, and local verifier	Fixed-budget MM-KWS-style reference implementation
Fitting data	seen_train	seen_train
Selection data	seen_cv	seen_cv
Approximate model size	4.68 million parameters	Reported separately in the reference-implementation record
Temperature	$(T_{\mathrm{TG}} = 2.0)$	$(T_{\mathrm{MM}} = 1.0)$
Trainable during Stage 4	No	No
Internal embeddings transferred	No	No
Parameters incorporated into PASEM-TR	No	No
Loaded during inference	No	No

Stage 4 objective weights

Loss term	Function	Weight
(L_{sup})	Supervised binary KWS loss	1.00
(L_{TG})	Frozen PASEM-AS posterior transfer	0.35

Loss term	Function	Weight
(L_{MM})	MM-KWS-style probability matching	0.25
(L_{rank})	Pairwise score-order preservation	0.10
(L_{query})	Query-level regularization	0.05

Note: Both teacher signals are frozen and used only during training. At inference, PASEM-TR uses only the trained Tibetan-PASEM student and the decision threshold defined by the evaluation protocol. The MM-KWS-style architecture, internal embeddings, and parameters are not incorporated into the Tibetan-PASEM inference pathway. These details are also stated in the response to Reviewer 1.

Table S2(c). Adaptation, optimization, and checkpoint records for the fixed-budget reference implementations

Item	MM-KWS-style	PhonMatchNet	Metric-UD-KWS fair-QBE
Original reference	MM-KWS [12]	PhonMatchNet [9]	Metric Learning for User-Defined Keyword Spotting [6]
Original query assumption	Text and spoken multimodal prompts	Phoneme-guided written query	Spoken enrollment/query by example
Querybank198 query input	Tibetan graphemic and approximate-phonological streams	Tibetan pseudo-phonological token sequence	Five spoken enrollment examples per query
Core retained	Multimodal fusion principle	Phonology-guided matching core	ResNet15 embedding and angular prototypical objective
Components reimplemented	Tibetan data loader, acoustic encoder, token embeddings, fusion layers, evaluator	Tibetan dataset adapter, tokenization, pair-level evaluator, threshold procedure	Querybank198 adapter, prototype construction, fair-QBE enrollment-exclusion audit
Omitted pathway	Original support-speech and AudioLM pathways	English G2P and Google speech-embedding pathways	None; spoken-enrollment role retained
Fitting data	seen_train query–speech pairs	seen_train query–speech pairs	Positive seen_train utterances
Negative sampling	3:1 negative-to-positive ratio	3:1 negative-to-positive ratio	Not applicable to prototype construction
Optimizer	AdamW	Adam	Adam
Learning rate	(2×10^{-4})	(1×10^{-4})	(1×10^{-4})
Weight decay	(1×10^{-4})	0	0
Batch construction	Batch size 8; gradient accumulation over 2 batches	Batch size 4	64 query classes × 2 examples
Training budget	10 epochs	10 epochs	10 epochs × 2000 steps

Item	MM-KWS-style	PhonMatchNet	Metric-UD-KWS fair-QBE
Auxiliary-loss weight	0.35	Not applicable	Not applicable
Spoken enrollment examples	None	None	5 per query
Random seed	2026	42	2026
Checkpoint-selection rule	Best seen_cv F1	Best seen_cv F1	Best seen_cv F1
Selected checkpoint	best.pt; the selected epoch number is unavailable in the released source snapshot	best.pt; the selected epoch number is unavailable in the released source snapshot	metric_ud_kws_best.pt / best.pt; the selected epoch number is unavailable in the released source snapshot
Threshold-selection data	seen_cv	seen_cv	seen_cv
Threshold rule	Maximize F1 subject to FAR ≤ 0.10	Maximize F1 subject to FAR ≤ 0.10	Maximize F1 subject to FAR ≤ 0.10
Held-out audio enrollment	No	No	Yes for queries absent from seen_train
Enrollment exclusion before scoring	Not applicable	Not applicable	Enrollment trials and trials sharing enrollment audio are removed
Evaluation role	Fixed-budget multimodal-prompt reference	Fixed-budget phonology-guided reference	Enrollment-based fair-QBE diagnostic
Convergence-equivalent tuning claim	Not claimed	Not claimed	Not claimed

Note: The three implementations use archived method-specific fixed budgets. Equal tuning effort and convergence-equivalent optimization are not claimed. The released records provide formal launcher settings, configuration files, command records, checkpoint filenames, selection rules, and score-generation audit summaries. Executed epoch-level validation histories, selected epoch numbers, checkpoint binaries, and raw baseline score archives were not present in the available source snapshots; no missing values were inferred.

Table S3. Query-level confusable-negative analysis, same-query control, and transcript-confirmed false-trigger coverage

Table S3(a). Aggregate query-level results on the cleaned confusable-negative condition

Item	Value
Model	PASEM-TR, seed 2028
Operating threshold	0.760016

Item	Value
Threshold-selection condition	cleaned seen_cv
Evaluation pairs	12,475
Positive pairs	2,081
Negative pairs	10,394
True positives	1,388
False positives	421
True negatives	9,973
False negatives	693
Micro Precision (%)	76.73
Micro Recall (%)	66.70
Micro F1 (%)	71.36
Micro FAR (%)	4.05
Micro FRR (%)	33.30
Macro query-average F1 (%)	67.95
Macro query-average FAR (%)	4.65
Macro query-average Recall (%)	64.55
Queries with zero Recall	0

Note: The threshold was selected on cleaned seen_cv and applied unchanged to all 12,475 cleaned confusable-negative pairs. The macro values are unweighted averages over the 20 query forms.

Table S3(b). Representative query-level results for PASEM-TR seed 2028

Tibetan query	Phonology source	Positive pairs	Negative pairs	F1 (%)	FAR (%)	Recall (%)	Error pattern
རིག གནས	hybrid_old_syllable_plus_rule_approx	40	200	42.25	8.00	37.50	Elevated FAR and low Recall
ཐག ཆོད	rule_based_wylie_approx	55	272	59.09	2.57	47.27	Low Recall
སྐད ཆ	inherited_exact_old_phonology	52	259	58.06	5.41	51.92	Moderate discrimination
གནས ཚུལ	inherited_exact_old_phonology	123	613	72.12	7.99	78.86	Elevated FAR
སྤྱི ཚོགས	hybrid_old_syllable_plus_rule_approx	234	1,162	73.61	2.32	64.96	Stable discrimination
སློབ གྲྭ	inherited_exact_old_phonology	187	937	82.93	3.10	81.82	Stable discrimination
ད ཅང	rule_based_wylie_approx	260	1,290	87.85	1.32	83.46	Stable discrimination
ཐག གཅོད	rule_based_wylie_approx	66	330	88.00	1.21	83.33	Stable discrimination

Note: Pair counts are integers; the remaining values are percentages. The eight cases include both higher-error and stable queries and are not intended to replace the full 20-query machine-readable results supplied in File S1. Error-pattern labels are descriptive summaries of the reported F1, FAR, and Recall values and are not causal phonetic diagnoses.

Table S3(c). Same-query ordinary-negative and confusable-negative controls

Condition	Queries	Shared positive pairs	Negative pairs	Precision (%)	Recall (%)	F1 (%)	FAR (%)
Ordinary-negative full pool	20	2,081	10,395	76.16 ± 3.22	68.11 ± 1.22	71.89 ± 1.71	4.29 ± 0.75
Confusable-negative full pool	20	2,081	10,394	76.63 ± 2.64	68.11 ± 1.22	72.10 ± 1.39	4.17 ± 0.62
Ordinary-negative count-matched	20	2,081	10,378	—	—	71.89 ± 1.71	4.29 ± 0.75
Confusable-negative count-matched	20	2,081	10,378	—	—	72.12 ± 1.38	4.17 ± 0.62

N*ote: Values are mean ± sample standard deviation across PASEM-AS seeds 2026, 2027, and 2028. Both full-pool conditions use the same 20 training-exposed query forms and exactly the same 2,081 positive pair keys. The count-matched subsets were selected deterministically before evaluation. For each seed, the threshold selected on cleaned seen_cv was applied unchanged to both negative conditions. The mean FAR difference between the full-pool targeted and ordinary conditions was −0.12 percentage points.*

Table S3(d). Transcript-confirmed false-trigger coverage underlying Figure 5

Item	Value
Model	PASEM-TR, seed 2028
Operating threshold	0.760016
Target queries	20
Total false-positive pairs	421
False-positive pairs containing at least one other exact Querybank written form	215
Transcript-mapping coverage (%)	51.07
Full relation-matrix dimensions	20 × 198
Displayed Figure 5 matrix dimensions	20 × 10
Relation unit	Unique false-positive speech segments
Matching rule	Exact contiguous Tibetan syllable-token sequence
Unique competing label assigned per negative pair	No

Note: A false-positive speech segment may contribute to multiple relation cells when several Querybank written forms occur in its transcript. The analysis is therefore a transcript-confirmed relation map rather than a unique-label multiclass confusion matrix. The complete 20 × 198 matrix, displayed 20 × 10 matrix, long-format relation records, per-query coverage totals, and score-to-transcript alignment audit are included in File S1.
